# Experimental investigation of thermophysical properties of Al_2_O_3_ TiO_2_ CuO Fe_3_O_4_ water ethylene glycol quadri hybrid nanofluid

**DOI:** 10.1186/s11671-026-04602-w

**Published:** 2026-04-28

**Authors:** Nasim Nayebpashaee

**Affiliations:** Department of Mining and Material Engineering, Tarbiat Modares University, Tehran, Iran

**Keywords:** Quadri-hybrid nanofluid, Thermophysical properties, Dynamic viscosity, Surface tension, Electrical conductivity, Temperature effect, Nanoparticle concentration

## Abstract

Multi-component nanofluids have attracted increasing attention for advanced heat transfer and energy systems; however, experimental data on oxide-based quadri-hybrid nanofluids, particularly regarding their coupled rheological and interfacial behavior, remain scarce. In this study, the thermophysical properties of a novel Al₂O₃–TiO₂–CuO–Fe₃O₄/water–EG quadri-hybrid nanofluid were experimentally investigated. The suspension was stabilized using oleic acid and sodium dodecyl sulfonate, and the nanoparticles were characterized by SEM and XRD analyses. Dynamic viscosity, electrical conductivity, and surface tension were measured in the temperature range of 298–340 K at nanoparticle volume fractions of 0.1–1%. The electrical conductivity increased monotonically with concentration, reaching a maximum enhancement of approximately 170% at 1% loading. Surface tension exhibited a non-monotonic U-shaped trend, with a maximum reduction of 29% at 0.1% concentration. Rheological analysis revealed two distinct regimes: a low-shear Newtonian plateau and a high-shear shear-thickening behavior, where viscosity increased by nearly one order of magnitude under combined high-temperature and high-shear conditions. To model this nonlinear behavior, a high-accuracy RBF-based correlation was developed, reproducing the experimental viscosity data with a deviation below 3%. The results highlight the strong coupling between temperature, shear rate, and nanoparticle concentration, providing new insights into the design of quadri-hybrid nanofluids with tailored flow and interfacial properties.

## Introduction

Nanofluids—colloidal suspensions of nanoparticles in conventional base fluids such as water, ethylene glycol, or oils—have attracted significant attention in recent decades due to their enhanced thermophysical performance compared to conventional heat transfer media [1, 2]. The incorporation of nanoparticles with high thermal conductivity into a base fluid improves important thermophysical properties, including thermal conductivity, viscosity, and specific heat capacity. In addition to thermal conductivity, dynamic viscosity, surface tension, and electrical conductivity are critical parameters that directly influence flow behavior, pumping power, and interfacial performance under varying temperature and nanoparticle concentration conditions. Consequently, nanofluids have been explored for applications in electronic cooling, solar thermal systems, heat exchangers, and advanced energy technologies [1–4].

Although single-component (monotype) nanofluids can improve heat transfer, their effectiveness is often limited by the intrinsic physical and chemical properties of the individual nanoparticles [1, 5]. To overcome these limitations, hybrid nanofluids, which contain two or more types of nanoparticles, have been introduced to exploit synergistic effects between the different materials. Such systems have demonstrated superior thermal conductivity, improved stability, and altered rheological behavior compared to single-component nanofluids [5–8].

A particularly promising development is the quadri-hybrid nanofluid, which combines four different types of nanoparticles. This multi-component approach offers an improved balance of thermal and flow properties relevant to advanced engineering applications, particularly in low-temperature and flow-sensitive systems [3, 7, 9].

Recent investigations have extended hybrid nanofluid research toward multicomponent and tetra-hybrid systems to further enhance thermophysical performance. Studies on tetra-hybrid nanofluids have reported improved thermal and flow characteristics compared to lower-order hybrid systems, particularly under complex flow conditions [10]. These developments reflect the growing interest in multi-nanoparticle formulations and their potential for tunable thermophysical behavior.

Recent experimental and theoretical studies support the superior performance of hybrid nanofluids [8, 11–17]. However, most previous studies have primarily focused on thermal conductivity enhancement, whereas systematic investigations of dynamic viscosity, surface tension, and electrical conductivity as functions of temperature and nanoparticle concentration remain comparatively limited. For example, Saghir and Rahman [11] reported improved forced convection in porous media using Al_2_O_3_–Cu and TiO_2_–SiO_2_ nanofluids, while Rashidi et al. [16] confirmed that increasing the volume fraction of nanoparticles significantly improves both thermal conductivity and viscosity, with temperature proving to be a critical factor. Kumar et al. [15] emphasized the influence of particle size, morphology, and composition of the base fluid on the stability and performance of nanofluids. Abdullah et al. [7] emphasized the importance of nanoparticle type and dispersion quality, while Ahmed et al. [17] demonstrated improved efficiency of solar collectors with dual-hybrid nanofluids. Kumar et al. [15] recently applied machine learning to model entropy generation in turbulent Al_2_O_3_–TiO_2_ nanofluids and achieved an improvement in heat transfer of more than 70%. Table 1 provides an overview of some studies on the thermophysical and rheological behavior of nanofluids.

**Table 1:** A review of recent experimental studies on hybrid nanofluids and their thermophysical properties

References	Nano additives	Base fluid	Volume fraction	Temperature range	Properties measured	Preparation method
[20]	Al_2_O_3_–ZnO–Fe_3_O_4_ (ternary)	Water	0.5–1.25%	25–65 °C	Thermal conductivity, viscosity, density	Two-step, surfactant-assisted mixing
[21]	MWCNT–TiO_2_–ZnO (ternary)	Water:EG (80:20)	0.1–0.4%	25–50 °C	Thermal conductivity	Two-step, ultrasonication, CTAB surfactant
[22]	Al_2_O_3_–ZnO–Fe_3_O_4_ (ternary)	Water	0.5–1.25%	25–65 °C	Thermal conductivity, viscosity	Two-step, no surfactant, ultrasonication
[23]	rGO^a^–Fe_3_O_4_–TiO_2_ (ternary)	Ethylene glycol	0.01–0.25 wt%	25–50 °C	Density, viscosity	Sol–gel method, ultrasonication, no surfactant
[24]	GO–TiO_2_–Ag and rGO–TiO_2_–Ag (ternary)	DI Water	0.0005–0.05 wt%	25–50 °C	Thermal conductivity, viscosity	Two-step: hydrothermal synthesis, bath & probe sonication

^a^Reduced graphene oxide

In addition, recent investigations have examined hybrid and multicomponent nanofluids under various thermal and flow conditions, including entropy analysis, Darcy–Forchheimer flow modeling, and oscillatory heated surface configurations. These studies demonstrated that nanoparticle combinations significantly influence transport characteristics and thermodynamic performance compared to mono-nanofluid systems [18, 19]. Such findings further confirm the importance of multicomponent formulations in tailoring thermophysical behavior under complex operating conditions.

As summarized in Table 1, most available experimental studies focus primarily on thermal conductivity and, to a lesser extent, viscosity measurements of ternary hybrid nanofluids. However, systematic investigations of surface tension and electrical conductivity remain notably absent in these reports. Moreover, quadri-hybrid oxide systems have not been experimentally explored under coupled variations of temperature and nanoparticle concentration. These limitations highlight the need for a comprehensive experimental characterization of multicomponent nanofluids, which involves the simultaneous evaluation of both rheological and interfacial properties.

Recent comprehensive reviews on hybrid nanofluids further emphasize the importance of preparation techniques, dispersion stability, and multidimensional thermophysical characterization beyond conventional thermal conductivity assessment [25, 26]. Experimental studies have also shown that nanoparticle morphology, composition, and concentration strongly influence rheological behavior and flow irreversibility in hybrid systems [27, 28]. However, despite these advances, systematic experimental datasets on oxide-based quadri-hybrid nanofluids, particularly including surface tension and electrical conductivity measurements under varying temperature and concentration, remain scarce.

Overall, these studies confirm that hybrid nanofluids, particularly those based on oxide nanoparticles, exhibit a significant enhancement in heat transfer performance. However, there are still other challenges, such as controlling viscosity, ensuring long-term dispersion stability, and understanding the complex interactions between the different types of nanoparticles.

Recent studies have further emphasized the importance of preparation and stabilization techniques for hybrid nanofluids, illustrating their influence on thermophysical properties and identifying key challenges in achieving stable and high-performance dispersions [26]. Concurrently, experimental studies have examined multi-nanoparticle hybrid systems (e.g., SiO_2_–MWCNT hybrid nanofluids) to investigate how nanoparticle combinations affect thermal behavior and flow characteristics beyond conventional single nanoparticle formulations [27, 28]. These works underscore the evolving interest in multicomponent formulations and support the need for further systematic experimental investigations of quadri-hybrid oxide nanofluids.

Recent studies in 2024 and 2025 have extensively investigated hybrid and ternary nanofluids under complex flow configurations, including rotating Darcy–Forchheimer flows and advanced conductivity models such as Yamada–Ota and Hamilton–Crosser correlations [29, 30]. These works primarily emphasize numerical modeling approaches using constitutive and machine learning-assisted techniques to predict thermal performance and flow behavior in hybrid and ternary nanofluids [29, 31–33]. Although such investigations provide valuable theoretical insight into hybrid nanofluid heat transfer mechanisms [31, 32], systematic experimental characterization of higher-order systems such as quadri-hybrid oxide nanofluids remains comparatively limited. In particular, experimental studies addressing the coupled effects of temperature, shear rate, and nanoparticle concentration on rheological and interfacial properties are still scarce, motivating the present work.

Although significant improvements have been reported for mono-, binary-, and ternary nanofluids, several limitations remain. Most previous investigations primarily emphasize thermal conductivity enhancement, while comprehensive rheological characterization under coupled temperature–shear–concentration conditions remains limited. In particular, experimental evidence on oxide-based quadri-hybrid nanofluids remains very limited in the open literature. Furthermore, existing studies often lack multidimensional predictive modeling capable of capturing nonlinear parameter interactions. Therefore, a systematic multidimensional experimental investigation combined with data-driven modeling is required to simultaneously characterize electrical conductivity, surface tension, and shear-dependent viscosity under coupled temperature–concentration–shear conditions.

The selection of Al_2_O_3_, TiO_2_, CuO, and Fe_3_O_4_ nanoparticles in the present study was not arbitrary but strategically based on their complementary physicochemical and transport characteristics. Al_2_O_3_ and TiO_2_ provide chemical stability and improved dispersion behavior, contributing to suspension robustness. CuO, with relatively higher intrinsic thermal and electrical conductivity among oxide nanoparticles, enhances charge transport and energy exchange mechanisms. Fe_3_O_4_ introduces magnetic responsiveness and interfacial polarization effects, potentially influencing microstructural dynamics and electrical conduction pathways under flow conditions. The synergistic combination of these oxides is intended to balance conductivity enhancement, dispersion stability, and controllable rheological response within a single multicomponent formulation.

Unlike mono-, binary-, or ternary nanofluids, the quadri-hybrid configuration provides additional degrees of freedom in tuning thermophysical and interfacial properties through multi-component synergy. This multivariate interaction may enable improved performance under coupled temperature–shear–concentration conditions, which remains largely unexplored experimentally.

Despite the growing number of studies on mono-, binary-, and ternary hybrid nanofluids, experimental investigations on oxide-based quadri-hybrid systems remain extremely scarce. In particular, systematic rheological characterization under coupled variations of temperature, shear rate, and nanoparticle concentration has not been previously reported for Al_2_O_3_–TiO_2_–CuO–Fe_3_O_4_ nanofluids dispersed in water–ethylene glycol mixtures. Understanding the coupled influence of temperature, shear rate, and nanoparticle concentration is crucial for real engineering systems, where nanofluids operate under dynamic flow and thermal conditions rather than static laboratory environments. The present study, therefore, introduces (i) a comprehensive experimental dataset covering electrical conductivity, surface tension, and shear-dependent viscosity under multidimensional parameter variation, and (ii) a high-accuracy RBF-based predictive model capable of capturing nonlinear coupling effects among temperature, shear rate, and nanoparticle concentration. The results demonstrate a temperature-induced transition from Newtonian to shear-thickening behavior, providing new insight into the complex rheological dynamics of quadri-hybrid oxide nanofluids.

## Materials and methods

### Materials

**Fig. 1:**
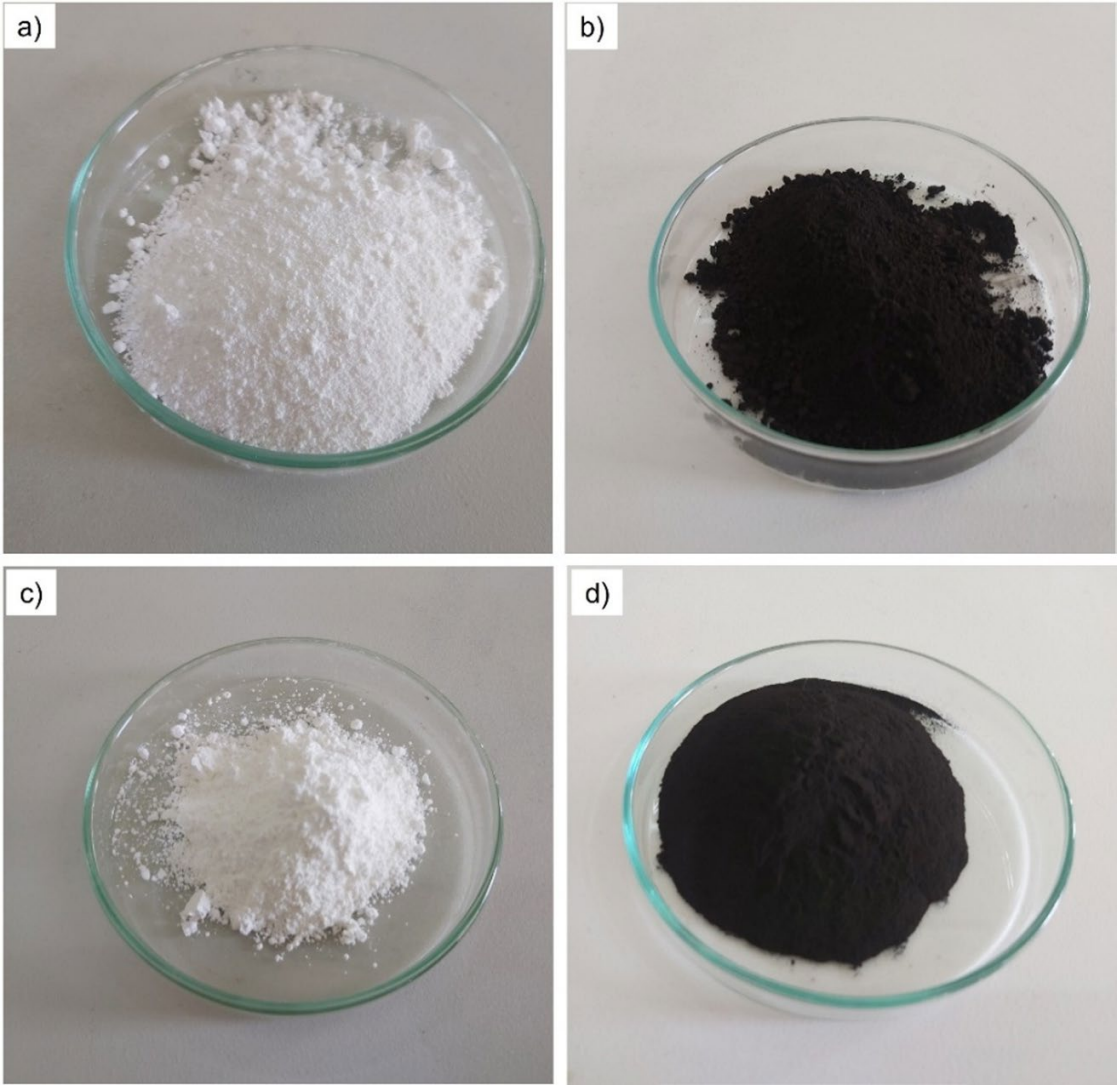
Nano powders used in the preparation of the quadri hybrid nanofluid:**a** Al₂O₃, **b** CuO, **c** TiO₂, and **d** Fe₃O₄

Four types of metal oxide nanoparticles—Al_2_O_3_, TiO_2_, CuO, and Fe_3_O_4_—were acquired from US Research Nanomaterials Inc. (USA). Their physicochemical properties, including average particle size, purity, specific surface area (SSA), morphology, density, and specific heat capacity, are summarized in Table 2, and their visual appearance is shown in Fig. 1.

**Table 2:** Specifications of nanoparticles used in this study

Parameter	Al_2_O_3_	TiO_2_	CuO	Fe_3_O_4_
Purity	+ 99%	+ 99%	+ 99%	+ 98%
Color	white	white	black	dark brown
Size	15 nm	21 nm	40 nm	30 nm
Morphology	Nearly spherical	Nearly spherical	Nearly spherical	Spherical
Specific surface area (SSA)	138 m^2^/g	220 m^2^/g	20 m^2^/g	50 m^2^/g
Density	3890 kg/m^3^	3900 kg/m^3^	6400 kg/m^3^	5000 kg/m^3^
Specific heat capacity	880 J/(kg.K)	523 J/(kg.K)	566 J/(kg.K)	148 J/(kg.K)

The base fluid was prepared by mixing ethylene glycol (EG) (analytical grade, purity ≥ 99%) with distilled water in a 1:1 volume ratio. The thermophysical properties of ethylene glycol are provided in Table 3.

**Table 3:** Characteristics of ethylene glycol

Characteristic	Value
Chemical formula	C_2_H_6_O_2_
Appearance	Clear, colorless liquid
Odor	Odorless
Molar mass	62.07 g/mol
Density	1113.20 kg/m^3^
Boiling point	197.3 °C
Thermal conductivity	0.244 W/m K (at 20 °C)
Viscosity	16.1 mPa·s (at 20 °C)

### Nanoparticle characterization

**Fig. 2:**
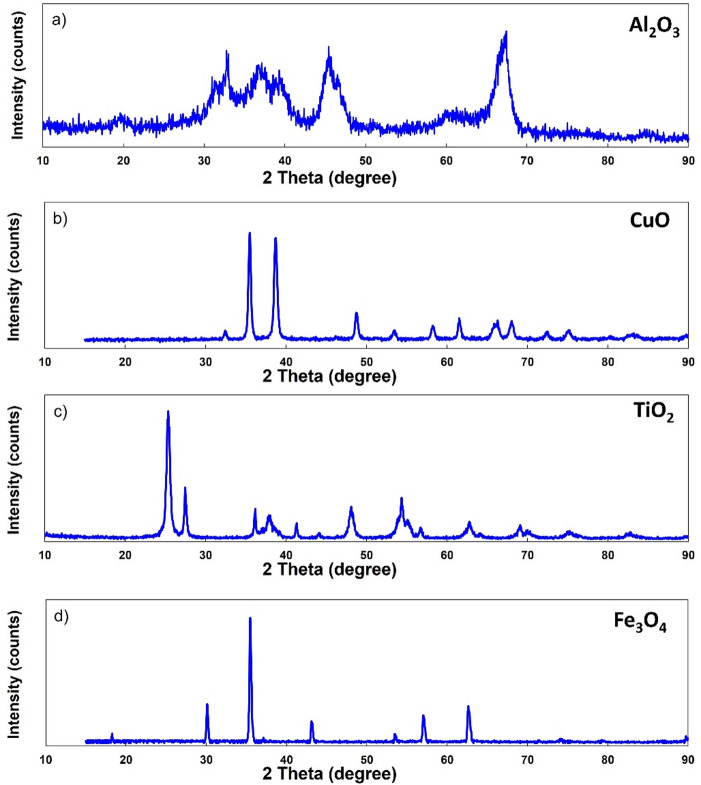
XRD patterns of the nanoparticles used in the quadri hybrid nanofluid: **a** Al₂O₃, **b** CuO, **c** TiO₂, and **d** Fe₃O₄. The patterns confirm the crystalline nature and phase purity of the individual nanoparticles

X-ray diffraction (XRD) analysis was conducted using a Philips PW1730 diffractometer with Cu Kα radiation (λ = 1.54060 Å), scanned from 2θ = 10° to 90° with a step size of 0.03° and a step scan time of 0.6 s, to determine the crystalline phases and structure of the nanoparticles. The XRD patterns of the individual Al_2_O_3_, CuO, TiO_2_, and Fe_3_O_4_ nanoparticles used in the preparation of the quadri hybrid nanofluid are presented in Fig. 2. The Al_2_O_3_ diffraction pattern (Fig. 2a) exhibits relatively broad peaks, suggesting a nanocrystalline structure with reduced crystallite size. In contrast, the CuO (Fig. 2b), TiO_2_ (Fig. 2c), and Fe_3_O_4_ (Fig. 2d) nanoparticles display sharp and well-defined peaks, suggesting high crystallinity. The observed diffraction peaks are in good agreement with the standard JCPDS data, confirming the phase purity of the nanoparticles.

**Fig. 3:**
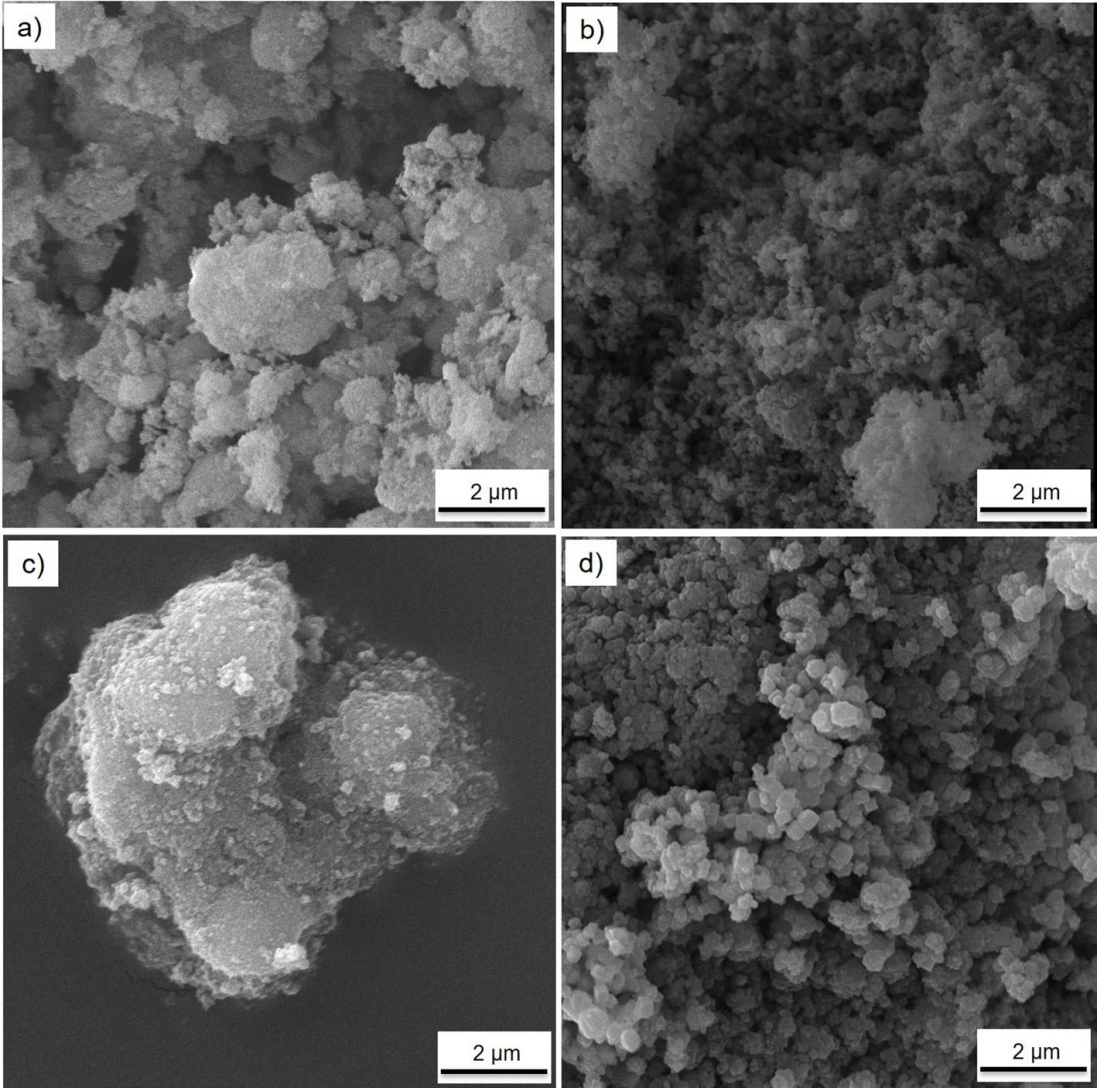
SEM micrographs of individual metal oxide nanoparticles used in the preparation of the quadri hybrid nanofluid: **a** Al₂O₃, **b** CuO, **c** TiO₂, and **d** Fe₃O₄

Surface morphology and particle size of the individual oxide nanoparticles were examined by scanning electron microscopy (SEM) using a TESCAN MIRA3 instrument. Before imaging, the powder samples were gold-coated using an IB-3 ionic sputter coater to improve surface conductivity and image resolution. The SEM images of Al_2_O_3_, CuO, TiO_2_, and Fe_3_O_4_ nanoparticles are shown in Fig. 3.

### Nanofluid preparation

A two-step method was used to prepare the quadri hybrid nanofluids, which included dispersing equal mass ratios of Al_2_O_3_, TiO_2_, CuO, and Fe_3_O_4_ nanoparticles into the water–ethylene glycol base fluid at solid volume fractions of 0.1%, 0.5%, and 1%. The required quantities were calculated using Eq. (1):

1 $$\varphi = \left[\frac{{\left(\frac{W}{\rho }\right)}_{Al2O3}+ {\left(\frac{W}{\rho }\right)}_{TiO2}+{\left(\frac{W}{\rho }\right)}_{Fe3O4}+ {\left(\frac{W}{\rho }\right)}_{CuO}}{{\left(\frac{W}{\rho }\right)}_{Al2O3}+ {\left(\frac{W}{\rho }\right)}_{TiO2}+{\left(\frac{W}{\rho }\right)}_{Fe3O4}+ {\left(\frac{W}{\rho }\right)}_{CuO}+{\left(\frac{W}{\rho }\right)}_{EG}+ {\left(\frac{W}{\rho }\right)}_{Water}}\right]\times 100$$

where φ is the nanoparticle volume fraction percentage, ρ is the density (kg/m^3^), and W is the mass (kg). To enhance dispersion stability, oleic acid (OA, 0.2 vol%) and sodium dodecyl sulfonate (SDS, 0.2 wt%) were added as surfactants. The relevant properties of OA are presented in Table 4.

**Table 4:** Characteristics of oleic acid

Characteristic	Value
Chemical formula	C_18_H_34_O_2_
Appearance	Clear, Pale yellow
Viscosity(@293.15 K)	38.80 mPa.s
Melting point	286.15 K
Freezing point	277.15 K
Cloud point (CP)	283.15 K ± 1
Pour point (PP)	273.15 K ± 1

The specific masses of nanoparticles, EG, and water used to prepare 1000 ml of nanofluid for various volume fractions are listed in Table 5.

**Table 5:** Mass of nanoparticles, Ethylene Glycol (EG), and water used for preparing a volume of 1000 ml of quadri hybrid nanofluid

Solid volume fraction (%)	Mass [± 0.001] (g)					
	TiO_2_	Al_2_O_3_	Fe_3_O_4_	CuO	EG	Water
0.1	0.975	0.972	1.250	1.600	556.043	498.606
0.5	4.875	4.862	6.250	8	553.817	496.609
1	9.750	9.725	12.500	16	551.034	494.114

**Fig. 4:**
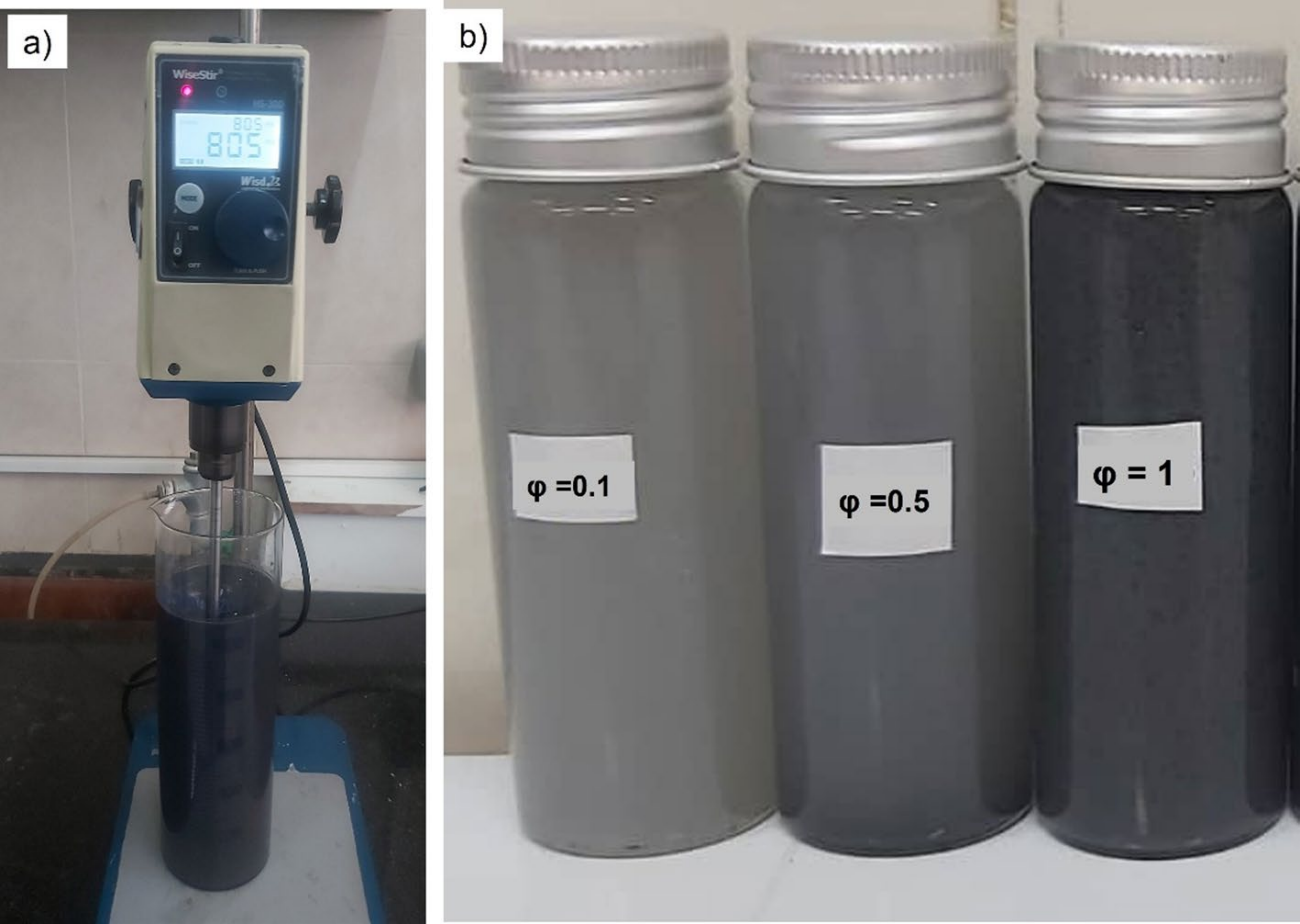
**a** Mixing process of quadri hybrid nanofluid using a digital stirrer at 800 rpm. **b** Samples with different nanoparticle volume fractions (φ = 0.1, 0.5, and 1 vol%)

The samples were initially stirred for 180 minutes with a magnetic stirrer (IKA RCT basic) and then dispersed ultrasonically for 4 hours. Subsequently, a digital stirrer (WiseStir HS-30, Fig. 4a) was used to further improve the dispersion. The appearance of the nanofluids at different concentrations (φ = 0.1, 0.5, and 1 vol%) is shown in Fig. 4b.

### Stability considerations of the quadri-hybrid nanofluid

The dispersion stability of the quadri-hybrid nanofluids was ensured through a combined electrostatic–steric stabilization strategy using sodium dodecyl sulfonate (0.2 wt%) and oleic acid (0.2 vol%), along with prolonged ultrasonication (4 h) following 180 min magnetic stirring.

SDS provides electrostatic repulsion between oxide nanoparticles in the polar water–ethylene glycol medium, whereas oleic acid contributes steric stabilization, particularly for Fe_3_O_4_ and CuO nanoparticles with relatively higher density.

The stability of the prepared suspensions was evaluated using the following criteria:

(i) Long-term visual sedimentation observation, which showed no visible phase separation or precipitation during the experimental period.
(ii) High repeatability of thermophysical measurements (viscosity, electrical conductivity, and surface tension), with deviations within experimental uncertainty.
(iii) Reproducible rheological responses, including a well-defined Newtonian plateau at low shear rates and consistent shear-thickening behavior at higher shear rates.

Each thermophysical measurement was repeated at least three times, and the reported values represent the average of the measurements. The maximum experimental deviation was within ± 3%.

It should be noted that quantitative stability assessments, such as zeta potential measurements or post-dispersion particle size analysis, were not performed in the present study. The focus was placed on the thermophysical characterization of freshly prepared suspensions under controlled laboratory conditions. However, the high repeatability of measurements and the absence of observable sedimentation during the experimental timeframe indicate adequate short-term dispersion stability for this investigation.

### Property measurements

#### Viscosity

**Fig. 5:**
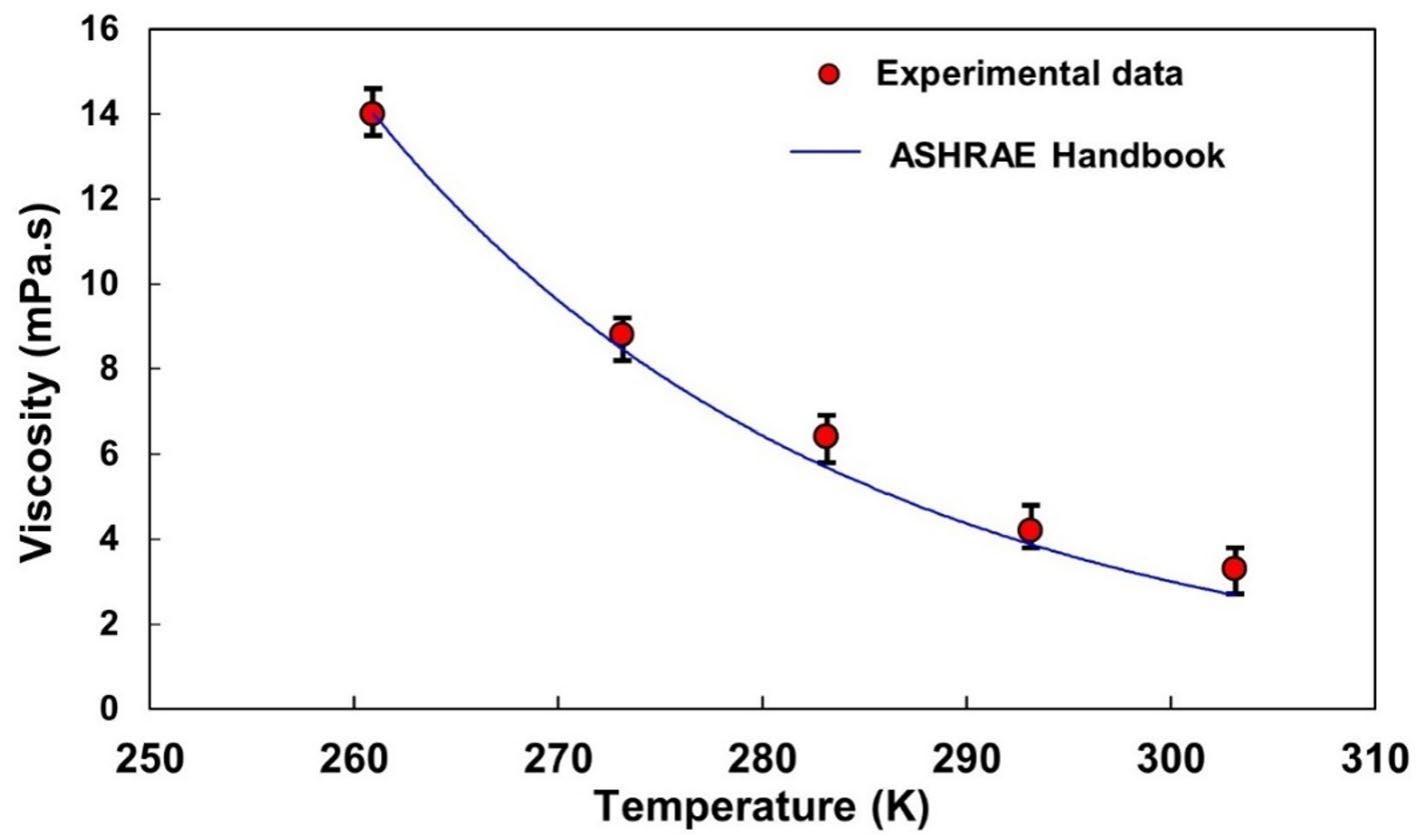
Calibration of the Brookfield DV2T viscometer with a 50:50 water–EC mixture: comparison of the experimental viscosity data with the reference values of the ASHRAE handbook [34], which shows a deviation of less than 2%

Viscosity was measured using a Brookfield DV2T viscometer over a shear rate range of 0.3–70 rpm and temperatures of 263.15–303.15 K for φ = 0.05–1%. The accuracy and resolution of the device were ± 0.1 and 0.2, respectively. The viscometer was calibrated with the base fluid (50:50 water-EG mixture), and the results were compared to the ASHRAE handbook [34] values, yielding a deviation of < 2% and confirming reliability (Fig. 5).

#### Electrical conductivity

Electrical conductivity was measured with a DDS-11A benchtop meter (range: 0–1.999×10^5^ μS/cm, accuracy ≤ ±1.5% F.S ±1 digit) at φ = 0.05–1% and T = 298 K. Calibration was performed with KCl standard solutions (1.41 and 12.88 μS/cm). Each test was repeated three times, and the average values were reported. The experiments were carried out at ambient pressure, a temperature of 25°C, and a humidity of ≤85%.

#### Surface tension

The surface tension was determined using a Surf-S1 tensiometer calibrated with acetone at 298.215 K. The measurements were performed at 297 K and repeated three times. The uncertainty was ± 0.1 mN/m.

### Uncertainty analysis

An uncertainty analysis was performed to evaluate the reliability and reproducibility of the experimental measurements. The uncertainties of the measuring instruments were obtained from manufacturer specifications and verified through calibration procedures (Table 6).

**Table 6:** Summary of Experimental Uncertainties

Property	Instrument	Calibration	Repeatability	Combined uncertainty
Viscosity	Brookfield DV2T	Base fluid vs ASHRAE	± 2%	± 3%
Electrical conductivity	DDS-11A	KCl standard	± 2%	± 1.5%
Surface tension	Surf-S1	Acetone reference	± 0.05 mN/m	± 0.1 mN/m

All thermophysical measurements were repeated at least three times under identical conditions, and the reported values represent the arithmetic mean. The maximum deviation between repeated measurements did not exceed ± 2% for viscosity and electrical conductivity and ± 0.05 mN/m for surface tension, confirming good repeatability.

For viscosity measurements, uncertainty sources included temperature control, rotational speed stability, and instrument resolution. The viscometer was calibrated using the base fluid (50:50 water–EG mixture), and the measured values were compared with ASHRAE reference data, yielding a deviation below 2%. The combined uncertainty of viscosity measurements was estimated using standard error propagation and was within ± 3%.

Electrical conductivity measurements were calibrated using KCl standard solutions, resulting in a measurement uncertainty of ± 1.5%, consistent with the device specification.

Surface tension measurements exhibited an uncertainty of ± 0.1 mN/m, primarily associated with temperature stability and device resolution.

The relatively low uncertainty levels and high repeatability confirm the reliability and scientific robustness of the reported thermophysical data.

## Results and discussion

### Electrical conductivity

The electrical conductivity of the Al_2_O_3_–TiO_2_–CuO–Fe_3_O_4_/water–EG quadri-hybrid nanofluid was measured at 298 K for various nanoparticle concentrations (Table 7).

**Table 7:** Measured electrical conductivity of the Al_2_O_3_–TiO_2_–CuO–Fe_3_O_4_ hybrid nanofluid in water–EG base fluid at 298 K as a function of nanoparticle volume concentration

Sample	φ = 0.1	φ = 0.5	φ = 1.0
Electrical conductivity (µS/cm) at 298 K	311	378	415

**Fig. 6:**
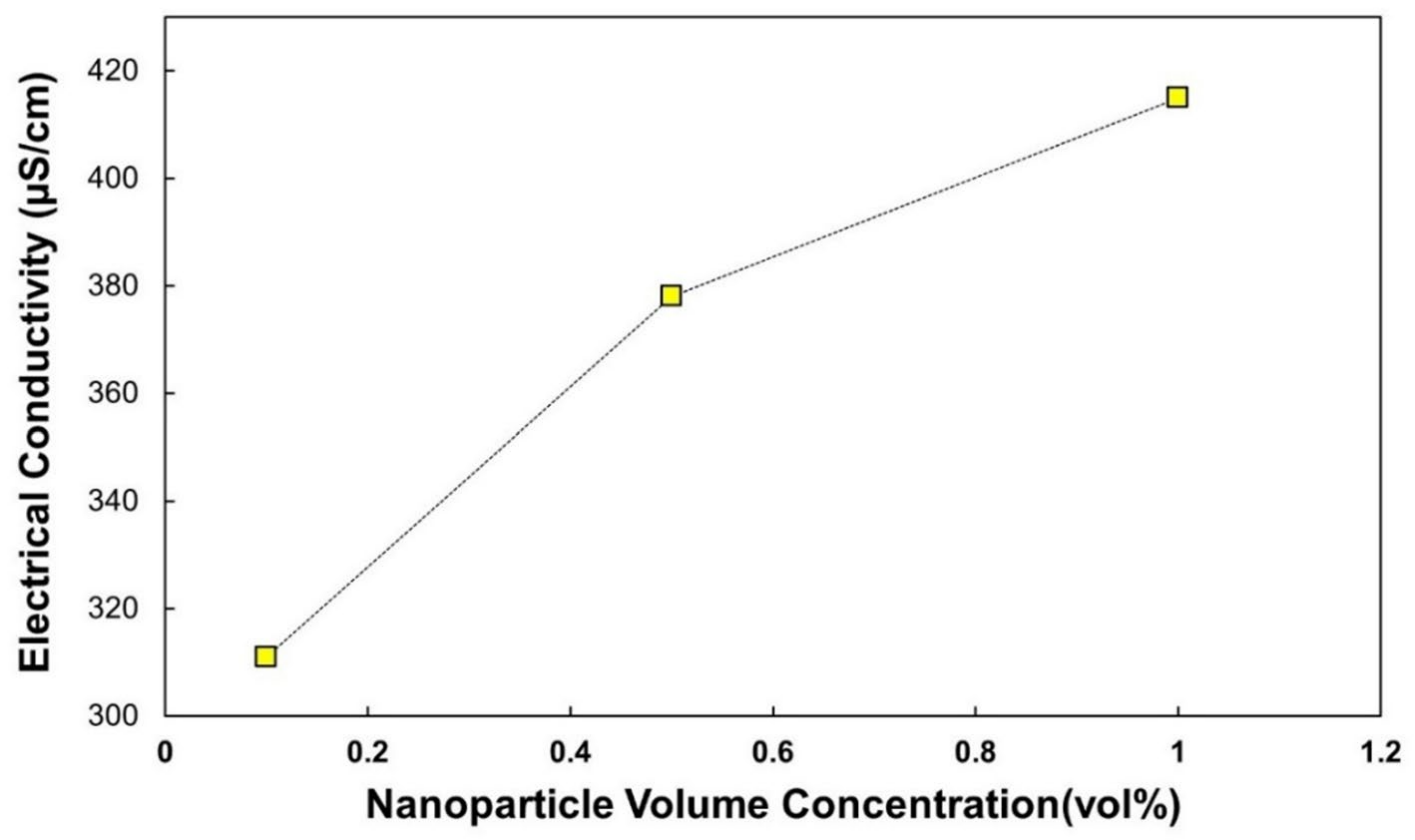
Electrical conductivity of Al₂O₃-TiO₂-CuO–Fe₃O₄/Water–EG quadri-hybrid nanofluid at 298 K as a function of the volume concentration of the nanoparticles. A monotonic increase is observed with increasing φ, which is attributed to synergistic electron transport mechanisms and improved dispersion stability

As shown in Fig. 6, the electrical conductivity increased steadily with nanoparticle concentration. This increase is due to the formation of additional conductive paths in the base liquid. Among the four oxides, CuO and Fe_3_O_4_ contributed the most due to their semiconducting and magnetic properties, which facilitate electron hopping, interfacial bilayer formation, and increased ion mobility. A similar mechanism was reported by Giwa et al. [12] for MWCNT-Fe_2_O_3_/water hybrid nanofluids, where the electrical conductivity improved by up to 1676%. In another study, Giwa et al. [35] observed that γ-Al_2_O_3_ MWCNT/water hybrid nanofluids showed an improvement in conductivity of up to 443% depending on the mixing ratio of the nanoparticles and operating temperature.

Although Al_2_O_3_ and TiO_2_ have a relatively low intrinsic conductivity, they play a decisive role in stabilizing the suspension. By preventing agglomeration of the particles and maintaining a homogeneous dispersion, they indirectly contribute to a higher conductivity. Kim et al. [36] also reported comparable stabilizing effects in graphene-MWCNT/solar glycol hybrid nanofluids, where the synergy of the nanoparticles ensured both an improvement in conductivity and long-term stability.

At higher concentrations (φ = 1.0%), a slower increase in conductivity was observed. This behavior can be explained by increased particle–particle interactions and a partial saturation of the conductive networks. According to percolation theory, once a critical threshold is reached, the overlap of conductive paths limits further enhancement in conductivity.

The monotonic increase in electrical conductivity with nanoparticle concentration can be physically explained by the formation of percolative conductive networks within the suspension. As φ increases, the interparticle distance decreases, facilitating electron hopping and interfacial charge transport, particularly between CuO and Fe_3_O_4_ nanoparticles. These oxides possess higher intrinsic electrical conductivity and contribute significantly to charge mobility.

In addition, interfacial polarization (Maxwell–Wagner–Sillars effect) occurs at the solid–liquid interface due to differences in dielectric properties between nanoparticles and the base fluid. Increasing nanoparticle concentration enhances the effective interfacial area, thereby strengthening polarization effects and contributing to conductivity enhancement.

At higher concentrations (φ = 1%), the reduced rate of conductivity increase suggests partial saturation of conductive pathways, consistent with classical percolation theory, where conductivity growth slows after reaching a critical network density.

Comparable enhancements have been reported in recent hybrid nanofluid studies; however, most available works have focused on binary or ternary formulations without experimentally investigating quadri-hybrid oxide systems under coupled concentration effects [37]. Similar conductivity enhancements have been noted in other experimental hybrid nanofluid systems [27, 28], where nanoparticle interactions and improved transport phenomena were identified as primary contributing factors.

Overall, the present results are consistent with previous reports on binary and hybrid nanofluids [12, 35, 36]. The synergistic interaction of Al_2_O_3_, TiO_2_, CuO, and Fe_3_O_4_ nanoparticles promotes the formation of interconnected conductive pathways and enhances interfacial charge transport within the suspension. In particular, the combined presence of semiconducting and magnetic oxides facilitates electron hopping and interfacial polarization mechanisms, leading to a substantial increase in electrical conductivity.

These findings demonstrate that quadri-hybrid oxide formulations enable controlled tailoring of electrical transport properties through nanoparticle synergy. Such tunable conductivity may be particularly beneficial in applications involving electro-thermal systems, sensors, and advanced heat transfer fluids where both electrical and thermophysical properties must be optimized simultaneously.

### Surface tension

**Fig. 7:**
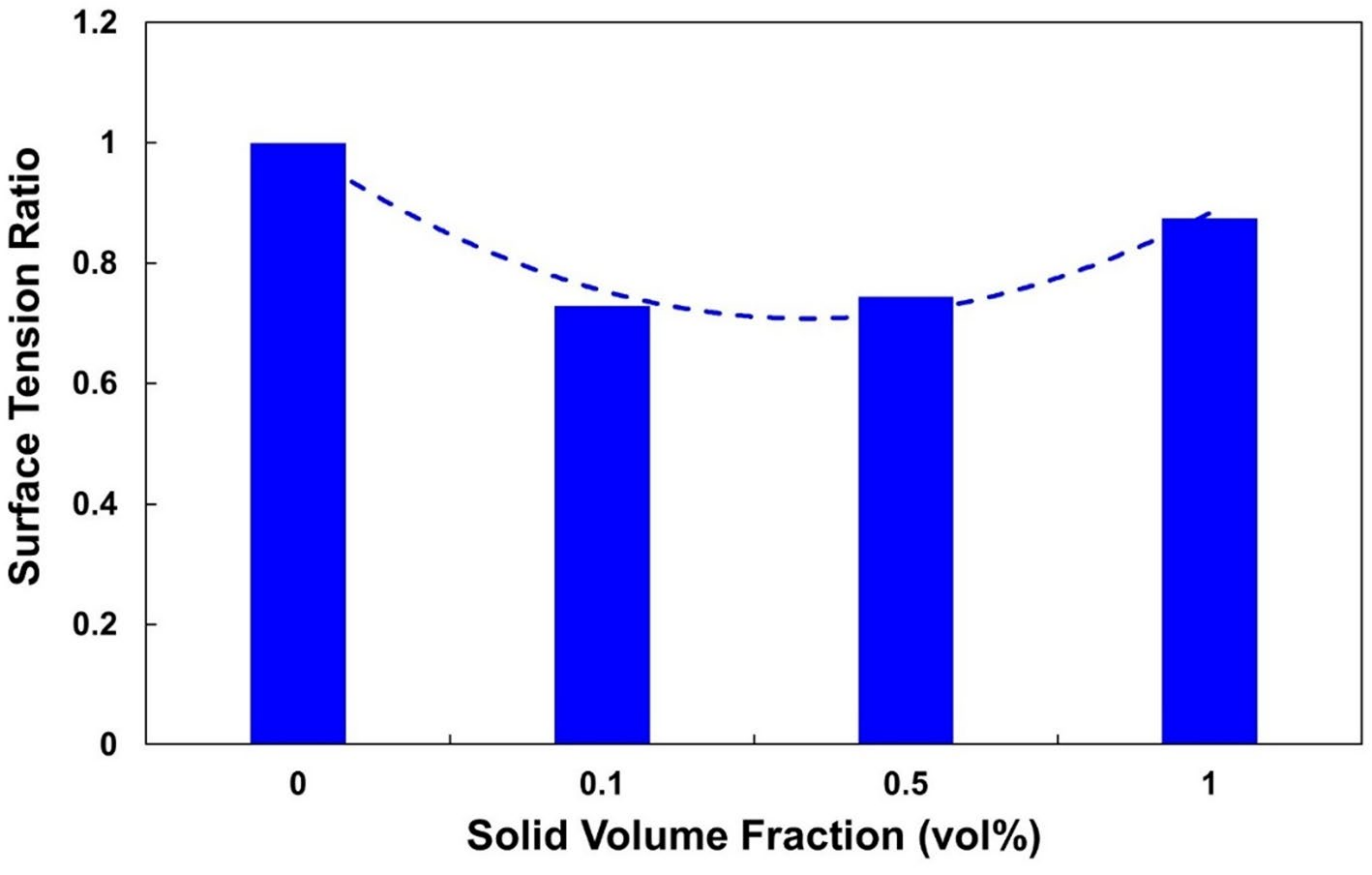
Variation of the normalized surface tension ratio (σ/σ₀) of the Al₂O₃-TiO₂-CuO–Fe₃O₄/water–EG-Quadri hybrid nanofluid at 297 K as a function of the solid volume fraction. A pronounced U-shaped behavior is observed, with the minimum occurring at φ = 0.1–0.5 vol%

The base fluid (water-EG, 50:50 v/v, stabilized with SDS and oleic acid) showed a surface tension of 40.14 mN/m at 297 K (Table 8). A significant reduction was observed after the addition of nanoparticles. At φ = 0.1%, the surface tension decreased to 28.49 mN/m (− 29.0%), and at φ = 0.5% it remained low (29.07 mN/m, − 27.6%). At φ = 1.0%, the surface tension partially recovered to 34.20 mN/m (− 14.8%). The normalized trend (σ/σ₀) is shown in Fig. 7.

**Table 8:** Surface tension of the quadri-hybrid nanofluid at 297 K

Sample	Base fluid	φ = 0.1	φ = 0.5	φ = 1.0
Surface tension (mN/m)	40.14	28.49	29.07	34.20

This U-shaped profile reflects the interplay of adsorption of the nanoparticles, interactions with surfactants, and aggregation of the particles. At low concentrations, the nanoparticles remain well dispersed and adsorb at the interface between liquid and air, disrupting the cohesive forces in the base liquid and reducing σ. At higher concentrations, particle aggregation reduces the adsorption efficiency, resulting in partial recovery.

The observed U-shaped trend in surface tension can be interpreted through the combined effects of surfactant adsorption and nanoparticle interfacial accumulation. At low concentration (φ = 0.1%), nanoparticles act as adsorption sites for surfactant molecules, enhancing interfacial packing and reducing surface free energy, which leads to maximum surface tension reduction.

As nanoparticle loading increases, a partial depletion of free surfactant molecules occurs due to adsorption onto particle surfaces. This reduces surfactant availability at the liquid–air interface, resulting in partial recovery of surface tension. Furthermore, increased particle–particle interactions at higher concentrations may reduce the efficiency of interfacial coverage, contributing to the observed non-monotonic behavior.

The results are consistent with contradictory literature reports [38–41]. For example, Zhou et al. [42] observed a monotonic decrease in σ for Al_2_O_3_/water nanofluids, while Banisharif et al. [43] reported an increase for Fe_3_O_4_/water-EG. These discrepancies illustrate the dependence of the surface tension on the type of nanoparticle, the composition of the base fluid, and the preparation method. These observations are consistent with previous studies reporting both monotonic and non-monotonic trends in the surface tension of oxide- and surfactant-stabilized nanofluids [39, 41–50].

Mechanistically, CuO and Fe_3_O_4_ — with higher polarity- promote adsorption and reduce σ, while Al_2_O_3_ and TiO_2_ improve dispersion stability. This synergy explains the pronounced reduction at φ = 0.1% and the moderate recovery at higher concentrations. Surfactants also play a key role: at low φ, SDS and OA adsorb strongly at the interface and lower the free energy (in accordance with the Szyszkowski isotherm [49]). At higher φ, however, the surfactants bind to the surfaces of the nanoparticles (OA to Fe_3_O_4_ and CuO; SDS to TiO_2_ and Al_2_O_3_), which reduces adsorption at the interface and increases σ.

From an application point of view, the reduced surface tension improves wettability, capillary-controlled flow, and heat transfer during boiling. The optimum concentration range for minimizing σ is 0.1–0.5 vol%, where the synergy between nanoparticles and surfactants is most effective.

### Shear flow behavior and viscosity of quadri hybrid nanofluids

To evaluate the viscosity of the Al_2_O_3_–TiO_2_–CuO–Fe_3_O_4_/water–EG quadri-hybrid nanofluid, it is first necessary to determine whether the suspension exhibits Newtonian or non-Newtonian behavior. Newtonian fluids follow a linear relationship between shear stress (τ) and shear rate ($\dot{\gamma }$) as given in Eq. (2):

2 $$\tau =\mu \dot{\gamma }$$

where μ is the apparent viscosity, τ is the shear stress, and $\dot{\gamma }$ is the shear rate. Non-Newtonian fluids deviate from Newton’s law, causing their shear stress to vary non-linearly with shear rate. The following equation demonstrates non-Newtonian behavior (Eq. 3):

3 $$\tau =m{\dot{\gamma }}^{n}$$

where n represents the power law index, and m represents the consistency index. When n < 1, the fluid shows shear-thinning behavior, whereas n > 1 indicates shear-thickening.

**Fig. 8:**
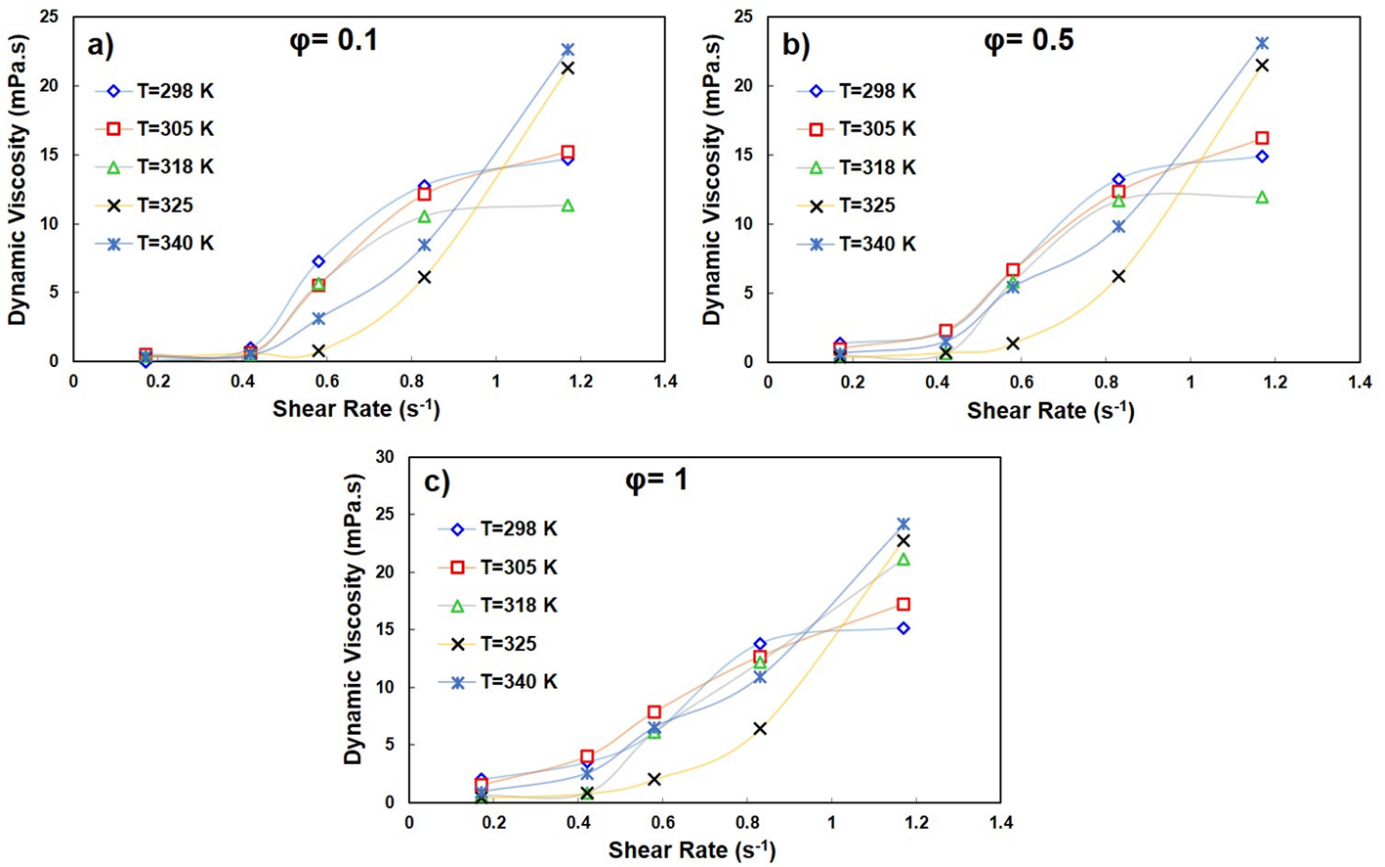
Viscosity of nanofluids against the shear rate for solid volume fractions of 0.1, 0.5, and 1 at different temperatures

Figure 8 shows the viscosity as a function of shear rate at nanoparticle concentrations φ = 0.1%, 0.5% and 1% at 298–340 K. At low shear rates ($\dot{\gamma }$ ≤ 0.3–0.4 s—^1^), the viscosity decreases monotonically with temperature, reflecting the thermal thinning of the water-EG base fluid. With increasing φ, the viscosity increases systematically due to stronger particle–particle and particle–fluid interactions. At higher shear rates ($\dot{\gamma }$ ≥ 0.5–0.8 s—^1^), however, shear thickening occurs. In particular, at $\dot{\gamma }$ ≈ 1.2 s—^1^ the viscosity at 340 K is almost twice as high as at 318 K for φ = 0.1–0.5% and ~ 60% higher than at 298 K for φ = 1%. This temperature-dependent reversal—from thermal thinning at low shear to thermal thickening at high shear- illustrates the competing influences of Brownian relaxation and hydrodynamic clustering, which is consistent with previous studies [51, 52].

**Fig. 9:**
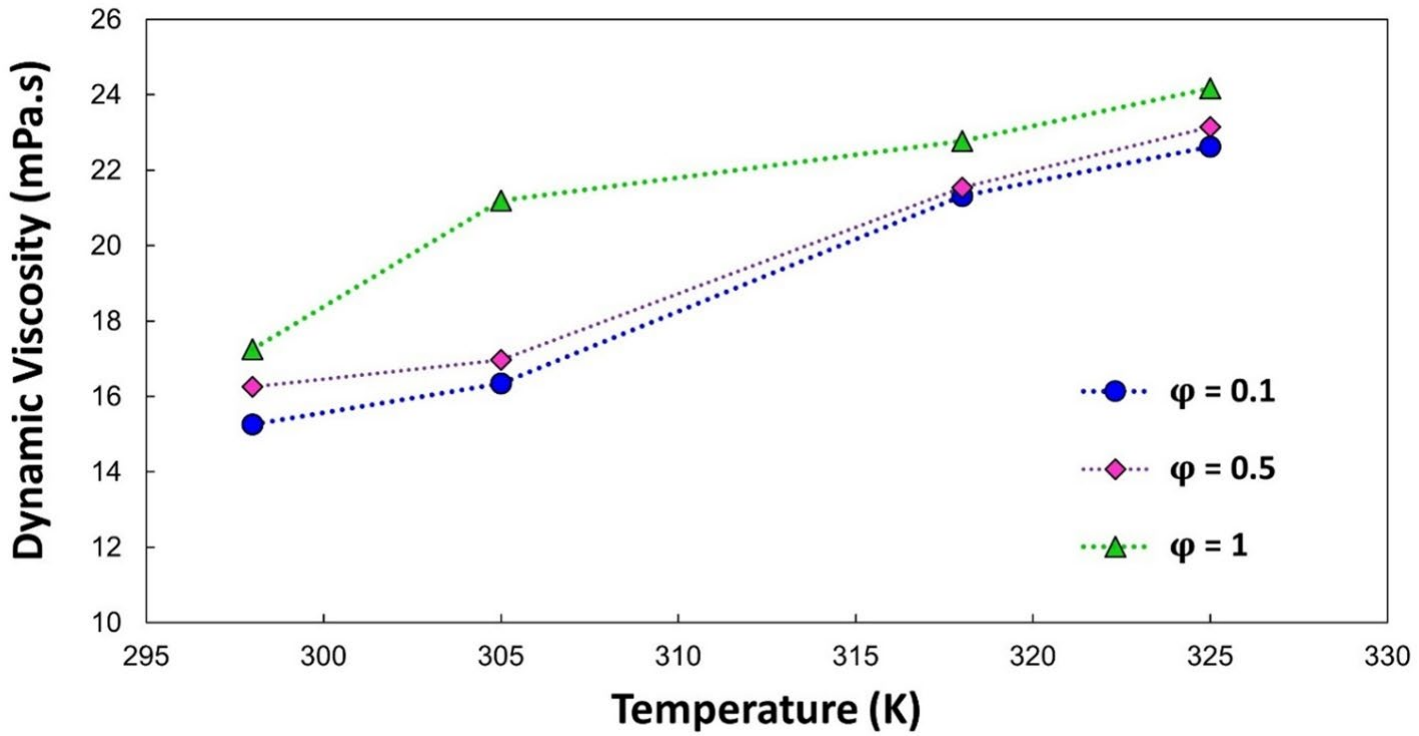
Experimental values of viscosity for various volume concentrations of nanofluids with respect to temperature

Figure 9 shows the viscosity-temperature trends at fixed concentrations. At low shear, the viscosity follows an Arrhenius-type thermal thinning law; for example, at φ = 1 %, the viscosity at 340 K is 50% lower than at 298 K. In contrast, the viscosity increases with temperature at high shear rates, which is due to increased particle collisions and the formation of hydroclusters that outweigh the dilution effect of the base fluid. Such reversals have also been reported for magnetite- and silica-based nanofluids [53].

**Fig. 10:**
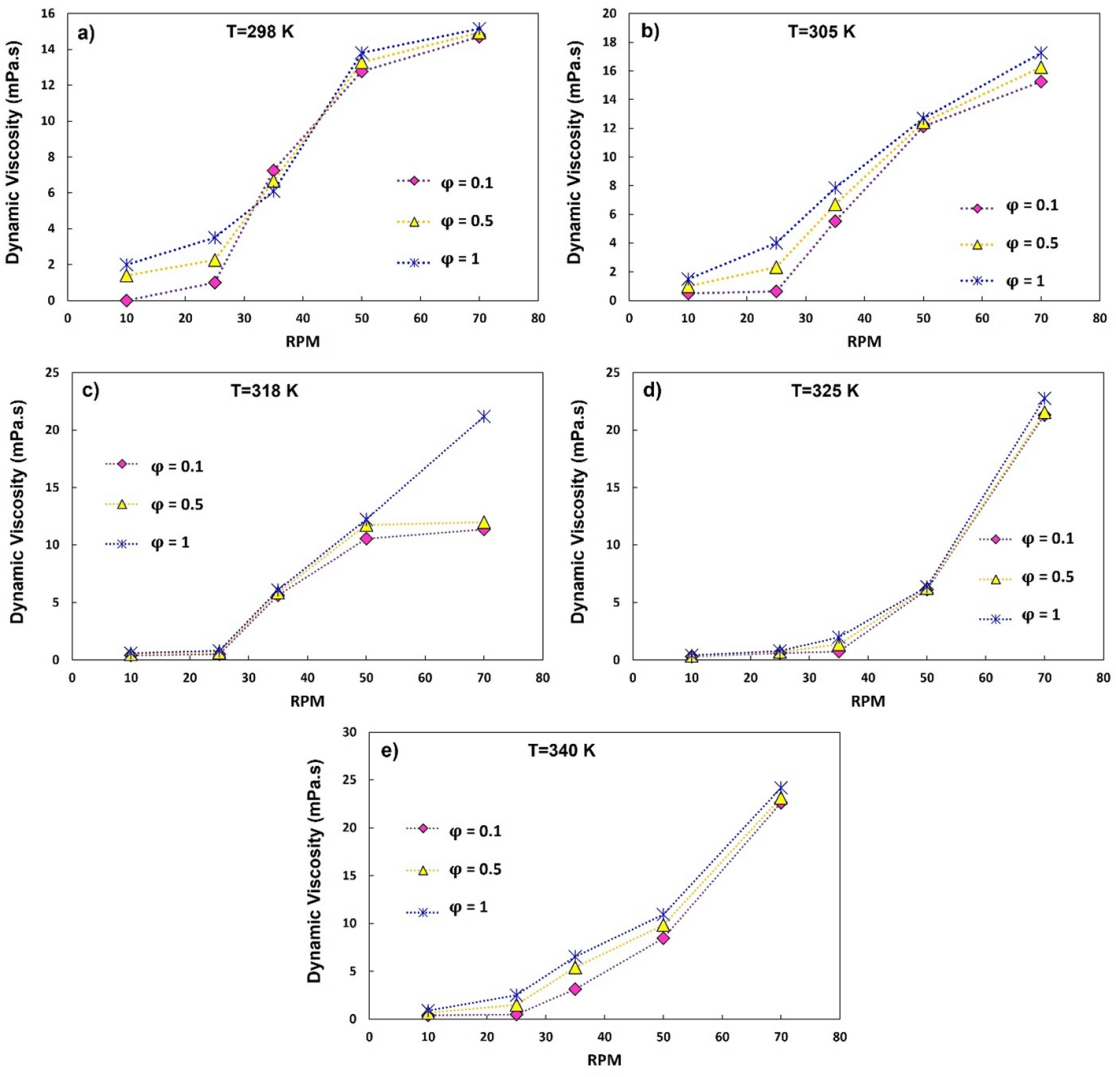
Viscosity of hybrid nanofluids versus the shear rate for various solid volume fractions at different temperatures

Figure 10 compares the dependence on the shear rate at different concentrations and fixed temperatures. At low shear rates, the viscosity increases monotonically with φ, as expected for colloidal suspensions. However, in the shear thickening regime, the effect of concentration becomes significantly weaker, with the curves for φ = 0.1–1% overlapping (differences of ~10–15%). This suggests that hydrodynamic clustering dynamically enhances the effective solids fraction and reduces the role of nominal concentration. A similar convergence has been reported for other shear-thickening suspensions [54].

Unlike many reported hybrid systems that primarily exhibit Newtonian or shear-thinning behavior, the present quadri-hybrid formulation demonstrates a temperature-dependent shear-thickening transition, which has rarely been documented in multicomponent oxide nanofluids [51, 52].

**Fig. 11:**
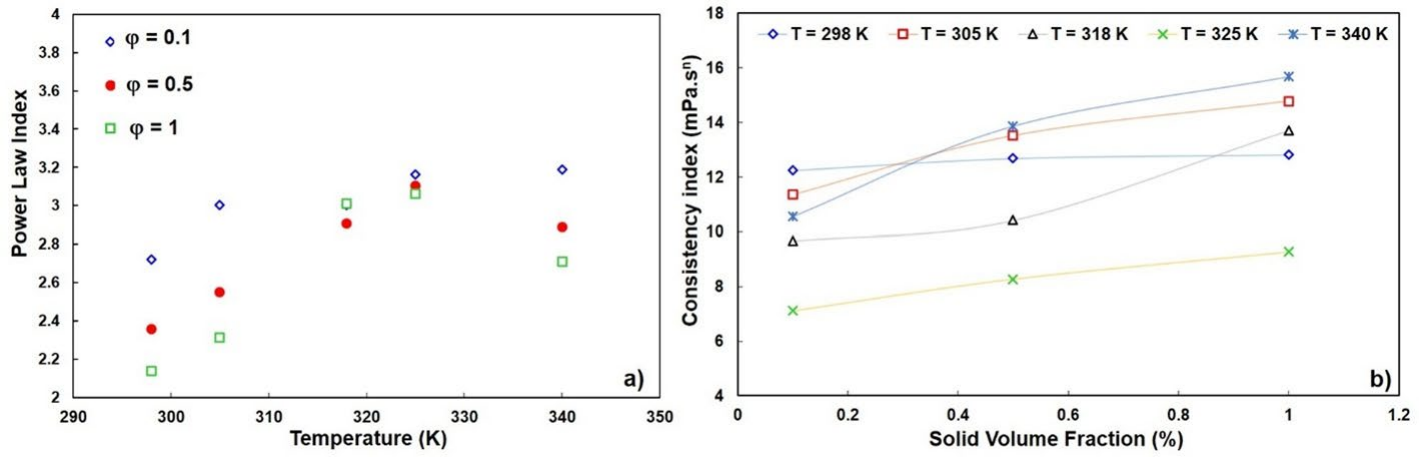
(a) Power law index and (b) Consistency index versus solid volume fraction at different temperatures

Figure 11 shows the power law fitting parameters: (a) the flow index (n) and (b) the consistency index (m). At low shear and low temperature, n ≈ 1, which corresponds to Newtonian behavior. With increasing φ and increasing temperature at high shear, n > 1, which confirms dilatant behavior. The consistency index m also increases systematically with φ, indicating an increase in base viscosity due to nanoparticle loading. These results are consistent with previous studies in which power-law models were applied to nanoparticle suspensions [55].

To summarize, the quadri-hybrid nanofluid exhibits two distinct regimes: (i) a low-shear Newtonian plateau, which is primarily determined by the temperature-dependent properties and concentration of the base fluid, and (ii) a high-shear and thickening regime, which is dominated by hydrodynamic clustering. At T ≥ 325 K and $\dot{\gamma }$ ≥ 0.8 s—^1^, the viscosity can increase by almost an order of magnitude compared to the low-shear plateau. Therefore, high shear viscosity data should be used in pump performance and heat transfer calculations, explicitly taking into account temperature-induced thickening.

The temperature and concentration dependence of viscosity results from competing microstructural mechanisms within the suspension. At low shear rates, Brownian motion dominates particle dynamics. Increasing temperature enhances Brownian diffusivity and reduces base fluid viscosity, leading to thermal thinning behavior.

However, at high shear rates and elevated temperatures, hydrodynamic clustering becomes significant. Increased particle collisions and reduced interparticle spacing promote the formation of transient hydroclusters, which increase resistance to flow and induce shear-thickening behavior.

The transition from Newtonian to shear-thickening behavior is therefore governed by the competition between Brownian relaxation forces and hydrodynamic stress-induced particle aggregation. Higher nanoparticle concentration increases effective volume fraction and interparticle interactions, amplifying this transition.

This multidimensional coupling between temperature, shear rate, and concentration explains the pronounced viscosity increase observed at T ≥ 325 K and $\dot{\gamma }$ ≥ 0.8 s⁻^1^.

This temperature-dependent rheological transition has important implications for pump design, thermal management systems, and flow-controlled energy applications where viscosity variations directly influence system efficiency. These findings demonstrate that neglecting shear-dependent and temperature-induced viscosity variations may lead to significant errors in pressure drop estimation and pump power calculations in practical systems.

To the best of the authors’ knowledge, no comprehensive correlation has yet been proposed for predicting the viscosity of Al_2_O_3_–TiO_2_–CuO–Fe_3_O_4_/water–EG quadri-hybrid nanofluids under simultaneous temperature, shear rate, and concentration variations. Accordingly, an accurate correlation based on experimental results to predict viscosity as a function of temperature, shear rate, and nanoparticle concentration is proposed in this study. This newly developed correlation shows acceptable accuracy in representing the measured data and is expressed as Eq. (4):

4 $$\mu =\sum_{i=1}^{N}{w}_{i}\,\,\mathrm{exp}[-\varepsilon {\Vert Z-{Z}_{i}\Vert }^{2}]$$

where

$$Z=\left[\frac{T-{\mu}_{T}}{{\sigma}_{T}} , \frac{\gamma -{\mu}_{\gamma }}{{\sigma}_{\gamma }} , \frac{\varphi -{\mu}_{\varphi }}{{\sigma}_{\varphi }}\right], \,\,\,\, Z=\left[\frac{{T}_{i}-{\mu}_{T}}{{\sigma}_{T}} , \frac{{\gamma}_{i}-{\mu}_{\gamma }}{{\sigma}_{\gamma }} , \frac{{\varphi}_{i}-{\mu}_{\varphi }}{{\sigma}_{\varphi }}\right]$$

Here, N corresponds to the number of experimental data points, w_i_ are the regression weights, ε is the kernel width parameter, while μ_T_, μ_γ_, μ_ϕ_, and σ_T_, σ_γ_, σ_ϕ_ denote the mean and standard deviation of the experimental dataset used for normalization. This ensures positivity of viscosity and high-fidelity interpolation.

The radial basis function (RBF) model successfully reproduced all experimental measurements with a maximum deviation < 3% and a negligible mean absolute deviation (~ 0%). This confirms the robustness of the correlation. In contrast to conventional semi-empirical models with fixed functional forms, the RBF approach adapts flexibly to multidimensional data structures and captures non-linear interactions between T, $\dot{\gamma }$, and φ. However, extrapolation beyond the analyzed ranges should be treated with caution.

Overall, the proposed correlation provides an accurate and generalizable description of viscosity in quadri-hybrid nanofluids. It can be directly integrated into computational fluid dynamics (CFD) simulations and process calculations, where reliable viscosity estimation is crucial for predicting flow and heat transfer performance.

### Comparative discussion with literature

To further evaluate the performance of the present quadri-hybrid nanofluid, the measured thermophysical properties are compared with selected experimental hybrid and ternary hybrid nanofluid studies in the literature. A detailed comparison is summarized in Table 9.

**Table 9:** Comparative overview of experimental hybrid and ternary nanofluid studies

References	Nanofluid system	Measured properties	Key observations
Giwa et al*.* [12]	MWCNT–Fe_2_O_3_/DI water	Viscosity & electrical conductivity	Conductivity ↑ (~ 1676%), viscosity ↑ (~ 35.7%) with concentration; T increase reduces viscosity and increases conductivity (experimental)
Zayan et al. [56]	GO–TiO_2_–Ag & rGO–TiO_2_–Ag (ternary)	Dynamic viscosity	Viscosity changes (~ 33–40%) with temperature and shear rate changes (experimental)
Sepehrnia et al*.* [57]	Fe_3_O_4_–TiO_2_–GO (ternary) in hydraulic oil	Dynamic viscosity	Viscosity measured across VFs and temperatures; Newtonian behavior with reduction at higher T (experimental)
Mousavi et al*.* [58]	CuO/CaCO_3_/SiO_2_ (ternary)	Viscosity & thermal conductivity	Viscosity increases with concentration; Newtonian behavior observed (experimental)
Present work	Al_2_O_3_–TiO_2_–CuO–Fe_3_O_4_/water–EG	Electrical conductivity, viscosity, surface tension	Electrical conductivity ~ 170% ↑; temperature-dependent Newtonian → shear-thickening regime; comprehensive dataset

In experimental work by Giwa et al. [12], hybrid nanofluids composed of multi-walled carbon nanotubes and Fe_2_O_3_ dispersed in deionized water exhibited increases in both electrical conductivity and dynamic viscosity with nanoparticle concentration (0.1–1.5 vol %). Increasing temperature reduced viscosity while enhancing electrical conductivity, with maximum enhancements of approximately 1676 % and 35.7 % for conductivity and viscosity, respectively, compared with the base fluid.

Ternary hybrid nanofluids such as GO–TiO_2_–Ag and rGO–TiO_2_–Ag have also been experimentally shown to exhibit temperature and shear-rate-dependent viscosity behavior; the measured viscosity changes by up to ~33 %–40 % when temperature and shear conditions vary [56]. Additionally, studies on binary CuO–TiO_2_ hybrid nanofluids reported typical viscosity increases with nanoparticle concentration and temperature effects on rheological behavior [59].

Sepehrnia et al. [57] investigated the dynamic viscosity of a ternary hybrid nanofluid based on Fe_3_O_4_–TiO_2_–GO in hydraulic oil over a range of temperatures and volume fractions, reporting Newtonian behavior with viscosity decreasing at elevated temperatures compared to the base fluid. This complements other studies on multi-nanoparticle systems and highlights the diversity of rheological responses in hybrid formulations.

Compared with the above experimental studies, the present quadri-hybrid Al_2_O_3_–TiO_2_–CuO–Fe_3_O_4_ nanofluid demonstrates simultaneous enhancement of electrical conductivity (~170 % at φ = 1 %) together with a temperature-dependent transition from Newtonian to pronounced shear-thickening behavior. While most reported hybrid and ternary systems exhibit predominantly Newtonian or shear-thinning responses, the multidimensional rheological transition observed here highlights the complex interparticle interactions in quadri-hybrid oxide formulations. Furthermore, unlike earlier works that typically focused on either conductivity or viscosity independently, the present study provides a coupled experimental dataset including electrical conductivity, surface tension, and shear-dependent viscosity under combined temperature–concentration variations.

These comparisons highlight the distinctive multidimensional thermophysical and rheological behavior of quadri-hybrid oxide formulations, showcasing their experimental contributions that extend beyond conventional binary and ternary hybrid systems.

## Conclusions

A systematic experimental investigation of the Al_2_O_3_–TiO_2_–CuO–Fe_3_O_4_/water–EG quadri-hybrid nanofluid was conducted under coupled variations of temperature, shear rate, and nanoparticle concentration. The major findings can be summarized as follows:

1. The electrical conductivity increased monotonically with nanoparticle concentration due to the formation of interconnected conductive pathways, with CuO and Fe_3_O_4_ playing a dominant role. A maximum enhancement of approximately 170% was achieved at φ = 1%.
2. The surface tension exhibited a non-monotonic U-shaped behavior. The maximum reduction (~ 29%) occurred at φ = 0.1%, attributed to synergistic interactions between surfactants and nanoparticles, while partial recovery at higher concentrations was associated with surfactant depletion and particle aggregation.
3. The viscosity results revealed two distinct rheological regimes:
  (i) a low-shear Newtonian plateau governed primarily by base fluid properties and nanoparticle loading, and (ii) a high-shear shear-thickening regime dominated by hydrodynamic clustering effects. At T ≥ 325 K and $\dot{\gamma }$ ≥ 0.8 s⁻^1^, the viscosity increased by nearly one order of magnitude compared to the low-shear plateau, demonstrating a temperature-induced rheological transition.
4. A high-accuracy radial basis function (RBF) correlation was developed to predict viscosity as a function of temperature, shear rate, and nanoparticle concentration. The proposed model reproduced the experimental data with a deviation of ≤ 3%, enabling reliable implementation in CFD simulations and engineering design calculations.
5. The combined experimental characterization and data-driven modeling provide multidimensional evidence of strong nonlinear coupling between temperature, shear rate, and nanoparticle concentration in quadri-hybrid oxide nanofluids. This comprehensive dataset establishes a reliable framework for predictive modeling and supports the rational design of advanced heat transfer and flow-sensitive thermal management systems.

Beyond the fundamental thermophysical insights, the observed multifunctional behavior suggests several potential engineering applications. The combined enhancement of electrical conductivity, tunable surface tension, and temperature-dependent rheological behavior makes the investigated quadri-hybrid nanofluid a promising candidate for advanced thermal systems. The improved electrical conductivity may benefit electro-thermal transport and electromagnetic-assisted heat transfer processes. The controllable surface tension can enhance wettability and boiling heat transfer performance in compact heat exchangers and microchannel configurations. Furthermore, the temperature-induced shear-thickening transition under elevated shear conditions indicates potential applicability in flow-regulated thermal management systems and smart cooling devices. The presence of Fe_3_O_4_ nanoparticles additionally enables the possibility of magnetically assisted flow control. Overall, these coupled thermophysical characteristics support the potential implementation of the quadri-hybrid nanofluid in solar thermal systems, electronic cooling modules, energy storage units, and aerospace thermal control applications.

## Data Availability

The data that support the findings of this study are available from the corresponding author upon reasonable request.
